# Dr. Trotter, on Citric Acid

**Published:** 1800-08

**Authors:** T. Trotter

**Affiliations:** Plymouth Dock


Gentlemen,

WITH much pleaſure I embrace the earlieſt opportunity to announce to the medical world, through the channel of your Journal, that I have ſucceeded in the cure of ſcurvy by the Citric Acid, in the *concrete form*, as prepared by Mr. Coxwell of Temple Bar, after the manner of Scheele.*

The hiſtory of health in the fleet for the laſt ſeven years has afforded us ſo large a ſtock of facts, that every information ſeems to have been obtained that could be wiſhed, for the effectual prevention and cure of ſcurvy; but there was ſtill a *deſideratum*. Some preparation of lemon juice was wanted, on a large ſcale, that could be preſerved in every climate for the longeſt voyage, and that ſecured all the antiſcorbutic virtues of this vegetable acid. Mr. Coxwell's preparation, in the form of cryſtals, poſſeſſes all theſe qualities: I alſo underſtand, that he can ſell it at a price lower than the common juice, as now ſupplied to the King's ſhips; and I can vouch, that its powers againſt this ſea diſeaſe, are equal to *any effect I have ever obſerved from the recent fruit in its moſt perfect ſtate*.

It is highly gratifying to myſelf, to find that the navy and commercial part of the country are now furniſhed with the laſt article that is neceſſary to preſerve the lives of ſeamen againſt a diſeaſe that is inſeparable from long voyages. Let me, however, remind the officer, the navigator, and merchant, that the crews of ſhips ought, neverthelefs, to be recruited, at as ſhort intervals as poſſible, with freſh meat and eſculent vegetables. I ſhould always like to ſee the lemon, in all its conditions, rather truſted in the cure, when we can do no

---

* Vide Crell's Journal for 1784.

no better. A long ufe of it weakens the digeftive powers, and impairs mufcular action; and thus adds another caufe to the lift of evils to which feamen are expofed, in bringing on that premature old age fo generally among them.

It takes from fixteen to eighteen parts of water to bring Mr. Coxwell's acid to the ftandard of lemon juice. The gums of the patient never bled after its firft adminiftration; they became florid on the third day; the colour of large livid blotches on the third day, alfo, were evidently different in their hue, and foft to the touch: other fymptoms declined in the fame proportion; and all this during the ufe of the fhip's common fare of falt meat, &c. in order to fhow its efficacy in the moft forlorn ftate, and where the difeafe was well advanced. We fhall, on fome future occafion, detail the firft cafes; where it will be obferved, that we have carefully avoided thofe errors into which others have fallen, on holding up new remedies againft fcurvy.

The mind of the philofopher will naturally offer fpeculations on this interefting fubject; that an acid, of vegetable origin, after undergoing the procefs of ebullition, combination with a calcareous bafe, and, at laft, difplaced by the fuperior attraction of another acid, fhould retain the fame effect on animal life, which it had in its recent form. But, "*we are fearfully and wonderfully made*, and here all conjecture muft ftop.

The medical readers of your Journal, who are connected with the mercantile world, will naturally extend this information. In the Eaft India fervice, and the Southern Whale Fifhery, fcurvy is a frequent difeafe. The concentrated ftate of this preparation of lemon juice, reduces the fhip load to the fize of a portmanteau. A befieged garrifon might be relieved by dropping from a balloon a few pounds of it, when fcurvy rendered it untenable, as happened with Fort St. Philip, at Minorca, laft war. I am,

Gentlemen,

Your moft obedient humble fervant,

T. TROTTER.

*Plymouth Dock,*

*July* 13, 1800.

P. S. Since finifhing this letter, I have been favoured with the following remark, by one of the refpectable and able furgeons who has been engaged in this inquiry. He fays, "I muft now confefs, that I have been not a little aftonifhed at the effects of the *concrete acid*, they have far furpaffed my moft fanguine expectations: the proportion of an ounce of *concrete falt* of lemon to a pint of water, as ufed by me, taftes ftronger of the *citric acid* than the common lemon juice.

juice. It is much more agreeable and palatable, being without any of that mufty tafte which the lemon juice always acquires."

*His Majefty's Ship, Superb,*

*July* 12, 1800.

T. WATHERSTON.

Mr. Watherfton alfo fays, that cafes of fcurvy, though with milder fymptoms, taking the common lemon juice from the fame date, continue on his lift, while the others are returned to duty.

---

